# How Culture and Trustworthiness Interact in Different E-Commerce Contexts: A Comparative Analysis of Consumers' Intention to Purchase on Platforms of Different Origins

**DOI:** 10.3389/fpsyg.2021.746467

**Published:** 2021-10-05

**Authors:** Anna Tikhomirova, Juan Huang, Shuai Chuanmin, Muhammad Khayyam, Hussain Ali, Dmitry S. Khramchenko

**Affiliations:** ^1^School of Economics and Management, China University of Geosciences, Wuhan, China; ^2^School of Mathematics and Physics, China University of Geosciences, Wuhan, China; ^3^English Language Department, Moscow State Institute of International Relations (MGIMO University), Moscow, Russia

**Keywords:** consumer behavior, purchase intention, trustworthiness, culture, e-commerce, context, country-of-origin

## Abstract

The outgrowth of e-commerce has advanced the development of countries' economies. Today, online marketplaces are targeting not only their local customers but are also spreading their interests overseas, expanding cross-border e-commerce. The current study aims to analyze the interaction of customer's personal traits, such as national culture, disposition to trust, and perceived trustworthiness, and their effect on the purchase intention within different e-commerce contexts. The contexts are chosen based on the country-of-origin parameter and serve as the moderator in the research model. Both direct and indirect effects of cultural dimensions on trustworthiness and purchase intention are analyzed within the research framework. The data for the analysis are randomly collected among the Russian population and assessed using structural equation modeling (SEM). The analysis results prove the marketplace context moderates the interaction of customers' personal traits among each other and their effect on the purchase intention. The study shows that dimensions of national culture have a more substantial effect on perceived trustworthiness and purchase intention in the Chinese marketplace context. The current study contributes to the analysis of customer behavior patterns within context, expanding context-related research direction. It increases the specificity of the culture and trustworthiness research and deepens the understanding of country-of-origin moderating effect in e-commerce. Moreover, addressing a high-level uncertainty avoidance culture within the research framework, the study diversifies the existing set of analyzed cultures in the e-commerce environment. The current study is applicable both in domestic and in cross-border e-commerce practice, broadening the understanding of consumer behavior patterns. The research model is relevant for the analysis of trust-effected behavioral outcomes.

## Introduction

In the globalized world, e-commerce is a dynamically developing business direction whose activities have erased borders, giving customers unlimited consumption opportunities. Countries with a developed e-commerce system are targeting overseas customers. With the shift of e-commerce from local to cross-border, it is essential not only to develop e-commerce inside the country but also to facilitate the development of ≪international online outshopping (IOO)≫ (Ramkumar and Jin, 2019). To fulfill this task, it is vital for the platforms expanding their business overseas to understand their potential customers' personal traits, which, on the one hand, will make consumption easy and attractive for customers and, on the other hand, will boost the overseas expansion of the marketplace.

Despite growing cross-border e-commerce, the customer perception of the foreign marketplaces has scarcely been analyzed. Some studies underline the importance of context and its effect on customers' behavioral outcomes. Ramaswamy (2013) advocates the importance of context and comprehension of customers as the fundamental principles of forward-looking businesses. Bansal et al. (2016) refer to the context and personality in relation to privacy concerns. The current study aims to fill the gap in the existing online context-related literature analyzing the influence of customer attributes, namely, the dimensions of the national culture following Hofstede's cultural dimension theory (Hofstede, 1980) and perceived trustworthiness (Mayer and Davis, 1995) in the context of marketplaces of different country-of-origin.

Russian consumers and their online purchase behavior are chosen as a subject for the analysis for several reasons. First, Russia is a country with a developing e-commerce infrastructure, while the analysis of consumer purchase intention in this kind of country is limited. Second, due to its size, population, and the growing number of Internet users, Russia is an attractive target for international e-commerce platforms. Third, Russian culture is characterized by an extremely high level of uncertainty avoidance (Hofstede, 1980). Uncertainty is a crucial point in the acceptance of e-commerce practices.

Based on the recent analytical data, three major groups of e-commerce platforms are dominating the market based on the country-of-origin: Russian, Chinese, and English speaking. The share of Chinese platforms has increased dramatically, from 25% in 2013 to 73% in 2019, while English-speaking platforms remain more or less stable, accounting for 21% in 2013 and 29% in 2019^1^. Despite having unlimited purchasing opportunities, customers still tend to refer to the local platforms, and the majority of purchases are made on Russia-based platforms. From each overseas group, the most popular platform is chosen for the analysis. AliExpress is taken as a representative of the Chinese e-commerce platform. In March 2021, the average daily number of local orders on the platform reached 64 thousand per day. The total number of unique buyers for the fiscal year exceeded 26.2 million people. The monthly audience of AliExpress is 29.1 million people. The daily audience is 8.8 million people^2^. E-bay is chosen as an example English-speaking platform. It started operating in Russia in 2010, launching a Russian language site in the attempt to expand its global presence. In the period from 2017 to 2018, the number of unique customers reached 850 thousand people^3^.

Obviously, as online consumption is not a common practice for the majority of the local population, consumers face a range of uncertainty issues. Among the most common issues are the inability to touch or try on the product and doubts about the product quality. Many customers prefer to pay for the goods on delivery, although overseas platforms mostly use a prepayment method. It is not straightforward for customers whom they should refer to in the case of a problem. They consider it is uneasy to return a product.

The research model is developed based on the existing theoretical literature. It assesses national culture on the individual level and customers' trustworthiness toward marketplaces of various country-of-origin. The study subjects customers' purchase intention of the goods online to the analysis, assessing the effect of the national culture and trustworthiness on it. The study attempts to answer the question of whether the effect of customer culture on trustworthiness dimensions, and the effect of perceived trustworthiness and cultural dimensions on purchase intention, differs throughout the context of foreign marketplaces.

Following originated by Bansal's et al. (2016) context-related research, the current study contributes to the research direction, increasing specificity of the culture and trustworthiness research. It addresses the country-of-origin concept, which is poorly analyzed in the e-commerce environment. Moreover, addressing a high-level uncertainty avoidance culture within the research framework, the study diversifies the existing set of analyzed cultures in the e-commerce environment.

## Literature and Hypotheses

The effect of the national culture on the consumers' behavior in general and e-commerce consumers, in particular, is difficult to argue (Lynch and Beck, 2001; Kacen and Lee, 2002; Gefen and Heart, 2006; Hallikainen and Laukkanen, 2018). Although the range of studies employing national culture is rather extensive, they are mainly concentrated on the particular group of cultures for the analysis majoring in the US and China. Park et al. (2012) add South Korea to the analyzed cultures. Chen et al. (2008) conduct a comparative analysis of virtual community members in China, Hong Kong, and Taiwan, which did not show any significant difference among the cultures. Capece et al. (2013) subject the Italian sample to the analysis. Hallikainen and Laukkanen (2018) uses the dimensionality of trust and trust-related constructs and their relationship to culture using China and Finland as samples. As can be seen, all the research works have been conducted in counties with well-developed e-commerce infrastructure. The current study aims to fill this research gap by analyzing the Russian customer, where e-commerce is still in the development stage and brings up many trust and uncertainty issues among the customers.

Several studies (Hofstede, 1980; Fukuyama, 1995; Doney et al., 1998) propose a close relationship between trust and culture. For example, Doney's et al. (1998) model incorporates cultural effects on trust-building processes. Even though Gefen and Heart (2006) advocates the necessity to use culture as a critical element in the analysis of e-commerce trust beliefs (trustworthiness), the US and Israel are chosen as the cultures for the analysis, which does not change the diversity of analyzed cultural sets in the e-commerce environment dramatically. Analyses of different cultural dimensions in the e-commerce environment have been conducted by Yoon and Kim (2009), Hwang and Lee (2012), Capece et al. (2013), Shiu et al. (2015), Shiu et al. (2015), and Hallikainen and Laukkanen (2018).

Taking Hofstede's cultural dimension theory, which initially consists of five dimensions, as the basis to assess culture on the individual level, the current study does not seek to employ all the cultural dimensions described in the theory as not all of them are considered to affect the trustworthiness dimensions of the customers in the online environment. Therefore, uncertainty avoidance and long-term orientation dimensions are subjected to the analysis.

Uncertainty avoidance (UAI), in which Russia scores exceptionally high (95) in general, refers to the tendency to avoid uncertain and new situations by members of a society (Hofstede and Hofstede, 2005). The second dimension subjected to the analysis is long-term orientation (LO), where Russia scores high as well (93) (Hofstede Insights).

It is supposed that the disposition to trust is not based on previous experience or hence on personal traits (Rotter, 1967; Kee and Knox, 1970). The range of studies advocates that representatives of different countries vary in their disposition to trust (Downes et al., 2002; Kirs and Bagchi, 2012). Some researchers suggest that disposition to trust, as a trust antecedent, mediates the relationship between personal characteristics, such as national culture and trusting behavior (Schoorman et al., 2007). The effect of the dimensions of the national culture on the disposition to trust has been assessed for Chinese and Finnish samples, where long-term orientation showed a statistically significant effect on disposition to trust, while uncertainty avoidance did not (Hallikainen and Laukkanen, 2018). Based on the previous findings, and supposing that culture subjected to the analysis in the current study differs from previously analyzed, it is hypothesized that:

H1: U*ncertainty avoidance affects disposition to trust online*
H2: L*ong-term orientation affects disposition to trust online*

Trust and its constructs are fundamental concepts of social interaction and have been thoroughly analyzed by scholars of different disciplines. In the e-commerce context, it plays a vital role and has been analyzed based on various aspects: disposition to trust as a factor of trust formation (Fukuyama, 1995; Gefen, 2000); perceived risk (Corbitt et al., 2003; Kim et al., 2008); perceived security (Bart et al., 2005; Casalo et al., 2007); perceived privacy (Bart et al., 2005; Casalo et al., 2007); perceived reputation (Doney and Cannon, 1997; Casalo et al., 2007); perceived usefulness (Davis, 1989; Pavlou, 2003); perceived system quality (Aladwani and Palvia, 2002; Yoon and Kim, 2009); perceived information quality (Cyr, 2008); perceived service quality (Parasuraman et al., 1988; Brown and Jayakody, 2009); design quality (Garrett, 2003; Bart et al., 2005); satisfaction (Anderson and Srinivasan, 2003, Chen and Chou, 2012); attitude (Fishbein and Ajzen, 1975; Teo and Liu, 2007); purchase intention (Kim et al., 2009); repeat purchase intention (Chiu et al., 2012); intention to use website (Gefen et al., 2003); and loyalty (Dick and Basu, 1994; Cyr, 2008).

Trust is considered to be a concept that develops over a long period of time, and as has already been mentioned, the research subjects to the analysis customers whose online experience is relatively new. Therefore, it seems necessary to address trustworthiness as a precedent of trust in the research framework.

Mayer and Davis (1995) is one of the first to differentiate two concepts: trust and trustworthiness. He refers to trust as the ≪willingness of a party to be vulnerable to the actions of another party based on the expectation that the other will perform a particular action important to the trustor, irrespective of the ability to monitor or control that other party≫ (Mayer and Davis, 1995, p. 712). Trustworthiness, according to Mayer and Davis (1995), is a characteristic of the trustee. It is the condition that leads to trust and consists of three dimensions. Later on, this classification was accepted and applied by a range of researchers (McKnight et al., 2002; Gefen and Heart, 2006; Lu et al., 2010; Bansal et al., 2016; Oliveira et al., 2017; Hallikainen and Laukkanen, 2018). Following Mayer's approach, the current study adopts three dimensions of trustworthiness: ability, benevolence, and integrity, and assesses customers' perception of the dimensions related to a particular platform. Ability is generally understood as the competence of the trustee to perform a task as supposed. Benevolence is the capability of the trustee to prioritize the truster's interests, putting aside his egocentric profit motives. Integrity supposes that the trustee acts according to a set of principles acceptable for the trustor (Mayer and Davis, 1995; Chen and Dhillon, 2003). In the e-commerce literature, customers' perception of the trustworthiness dimensions is subjected to the analysis.

Disposition to trust is a well-established influencer on trustworthiness dimensions. Previously, several studies subjected the disposition to trust to the analysis in the online environment and found a positive correlation between it and trustworthiness dimensions (Gefen et al., 2003; Teo and Liu, 2007; Kim et al., 2008; Lu et al., 2010; Hallikainen and Laukkanen, 2018).

*H3: Disposition to trust affects consumers' belief in the ability of the platform*
*H4: Disposition to trust affects consumers' belief in the integrity of the platform*
*H5: Disposition to trust affects consumers' belief in the benevolence of the platform*

The relationship of the national culture and perceived dimensions of trustworthiness has been analyzed in a range of research works (Gefen and Heart, 2006; Yoon and Kim, 2009; Hwang and Lee, 2012; Hallikainen and Laukkanen, 2018).

For the online environment, UAI is crucial, having a significant impact on trustworthiness (Hwang and Lee, 2012; Shiu et al., 2015). Hwang and Lee (2012) found a moderating effect of UAI on integrity and ability, while benevolence did not show any relation with national culture or purchasing intention. Yoon and Kim (2009) claims that UAI is the most significant dimension of the national culture, which not only affects the acceptance of new technology, particularly e-commerce activities, but also shows a significant effect on behavioral outcomes as well as trustworthiness. Hallikainen and Laukkanen (2018) confirm these findings.

**H6:** *Uncertainty avoidance affects consumers' belief in the ability of the platform*
**H7:** *Uncertainty avoidance affects consumers' belief in the integrity of the platform*
**H8:** *Uncertainty avoidance affects consumers' belief in the benevolence of the platform*

It is believed that in the e-commerce environment, members of a high long-term-oriented society require a deep level of trust. It is essential for them to be familiar with the online platform before purchasing (Harris and Dibben, 1999). Hallikainen and Laukkanen (2018) found out that LO affects the disposition to trust and demonstrates a complementary mediating effect on all the dimensions of trustworthiness.

**H9:** *Long-term orientation affects consumers' belief in the ability of the platform*
**H10:** *Long-term orientation affects consumers' belief in the integrity of the platform*
**H11:** *Long-term orientation affects consumers' belief in the benevolence of the platform*

All the dimensions of trustworthiness may differ independently and are considered a dynamic phenomenon. In their models, Mayer and Davis (1995) and Rousseau et al. (1998) use trust as a mediator to explain the relationship between trustworthiness and behavioral outcome (purchasing intention in the context of the current study). Colquitt et al. (2007) suggests that trustworthiness may be significant without the mediating role of trust. All the elements are proven to have a significant effect on the behavioral outcome and are used as predictors of affective commitment as well as trust. Following previous studies, trustworthiness dimensions are adopted and used in the research framework without the mediating role of trust. It has been assumed that trustworthiness dimensions can potentially diminish the level of uncertainty that is crucial for online purchase intention (Gefen and Straub, 2004). Prior studies show that dimensions of trustworthiness affect different online behavior (Gefen, 2002).

In the online environment, Gefen and Heart (2006) indicate that integrity primarily affects intentions to make a purchase, while ability primarily affects intentions to inquire about the product without actually purchasing it. Pavlou and Dimoka (2006) analyze the effect of trustworthiness on price. The current study assesses whether dimensions of perceived trustworthiness affect purchasing intention in the different contexts of online platforms.

The correlation between the perceived ability and purchase intention was found prior in e-commerce-related research literature (Gefen, 2000; Colquitt et al., 2007; Lu et al., 2010; Hwang and Lee, 2012). Therefore, it is hypothesized that:

*H12: Consumers' belief in the ability of the platform affects purchase intention*

Hwang and Lee (2012) claim that there is no relationship between benevolence and purchase intention. However, Colquitt et al. (2007) argue that there is a minor unique relationship between benevolence and behavioral outcome.

*H13: Consumers' belief in the benevolence of the platform affects purchase intention*

Several scholars found a positive effect of integrity on purchasing intention (Gefen, 2000; Colquitt et al., 2007; Hwang and Lee, 2012)

***H14***: *Consumers' belief in the integrity of the platform affects purchase intention*

According to Gefen and Heart (2006), disposition to trust, along with other trust-related measurements, affects actions within the e-commerce platform, including purchase intention.

*H15: Disposition to trust affects customers' purchase intention*.

A comprehensive literature analysis has shown that the majority of studies devoted to trustworthiness and culture in the e-commerce environment mostly ignore the context, assuming that trust always positively affects the behavioral outcome (Gefen and Heart, 2006). The study follows Pavlou and Gefen (2005) suggestion that e-commerce context moderates the effect of trust, which, in its case, affects the behavioral outcome. The current research compares two e-commerce contexts: Aliexpress and eBay, supposing that trustworthiness in these contexts may vary. The study differentiates context based on the country-of-origin. The concept of country-of-origin in its application to the purchasing intention and customer preferences has been widely analyzed in an offline environment. Dichter (1962) states that the concept “Made in.” significantly affects the consumers' acceptance of the product. Elliot and Cameron (1994) claim that country-of-origin influences the perception of the product by the consumer, placing it as one of the influential factors affecting consumer purchase intention. Hong and Wayer (1989) advocate that the country of origin indirectly affects customers' procession of information about the product. The application of this concept in e-commerce is relatively limited. Ramkumar and Jin (2019) analyze pre-purchase intention and post-purchase consequences of IOO using the effect of E-tailer's country image as a moderator factor, proving that country image moderates the relationship between transaction utility and IOO intention.

The current study suggests that country-of-origin moderates the effect of the national culture on the dimensions of trustworthiness toward the e-commerce marketplace and purchase intention in two marketplaces.

Based on the geography of the IOO of the customers subjected to the analysis, two online platforms were chosen as examples. The first one, Aliexpress, is a global e-commerce platform that provides goods produced mainly in China. It started operating in 2010 and can currently be considered a B2C and C2C platform. Today, the platform operates in English, Russian, Spanish, Arabic, and several other languages, alleviating language barriers and easing uncertainty issues. For Russian online customers, Chinese platforms are occupying the second favored place. Among Chinese platforms presented on the Russian market, Aliexpress is the most popular, while Russia is the largest market for AliExpress. From a customer perspective, trust issues on this platform can be related to machine translation errors, difficulty in communication with suppliers, quality issues, long delivery period, and country-of-origin image.

The second platform is eBay. It is an American-based company providing services in the area of online auction and online stores. From 2010, eBay started its international operation, and customers were able to use it in the Russian language. In 2012, it opened its branch in Russia. Despite the ability to use the website in the Russian language, where machine translation was not particularly accurate, there are two main drawbacks for the expansion of eBay in Russia, specifically the usage of PayPal, which Russian users are not familiar with, and logistics problems.

## Materials and Methods

### Research Model

**Figure 1:**
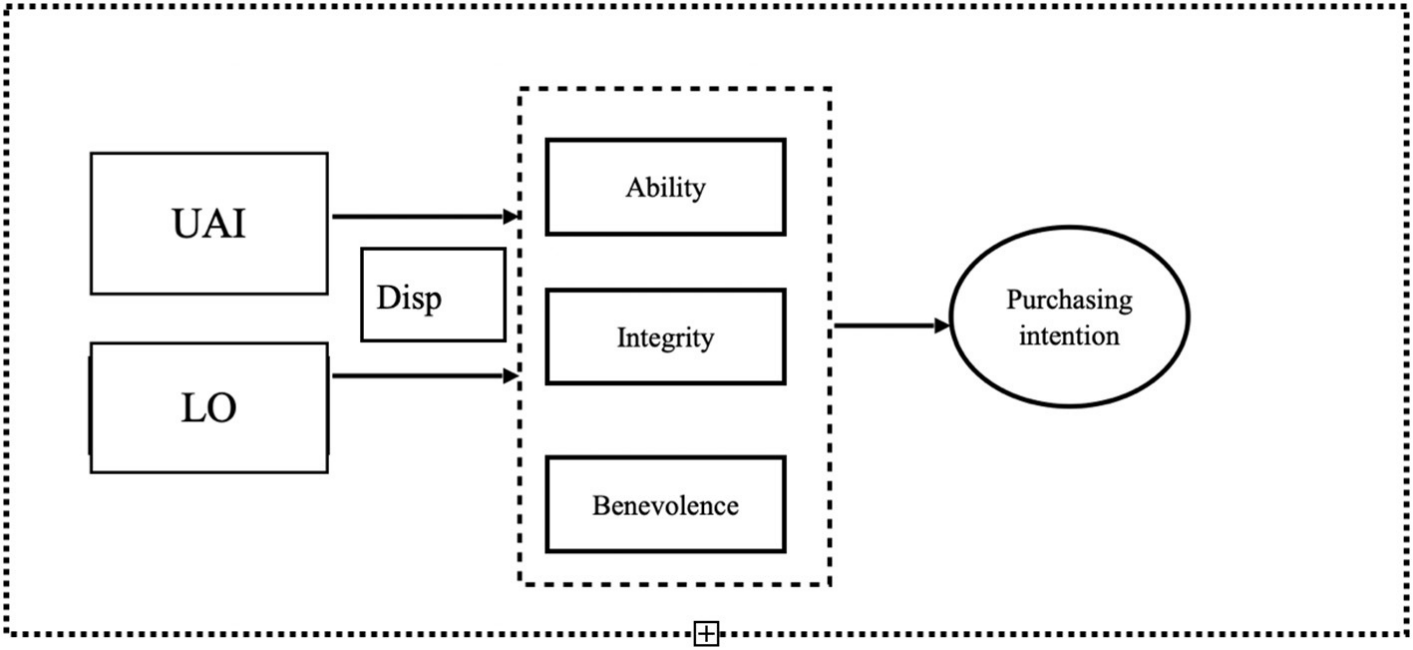
Research model.

Based on the analysis of the existing theoretical literature, the following research model is developed. It consists of four parts. Two dimensions of the national culture measured at the individual level, adopted from Hofstede's cultural dimension theory, are used as independent variables; three constructs of trustworthiness adapted from Mayer and Davis (1995) are mediator factors in the model. It is supposed that disposition to trust mediates the relationship between the cultural dimensions and trustworthiness, nevertheless not excluding the possibility of direct effect between cultural dimensions and trustworthiness. Purchase intention is taken as a dependent variable. It is hypothesized that e-commerce platforms moderate the effect of the national culture and trustworthiness on purchase intention, so the platform is used as a moderator in the research model presented in Figure 1.

### Research Methodology

Based on the existing research literature, the following approach is developed. In order to comprehend customers' culture at the individual level, Hofstede's cultural dimension theory is used as the basis. The theory assessed national culture in five dimensions. Meanwhile, the current research adopts two dimensions for the analysis: uncertainty avoidance and long-term orientation, as the most relevant to the trustworthiness issue. The rest are excluded as irrelevant for the current study.

As trustworthiness is used as a mediator factor in the conceptual model of the research, three dimensions of trustworthiness are assessed: ability, integrity, and benevolence as the most commonly used in trust-related research works (Serva et al., 2005; Colquitt et al., 2007). Disposition to trust, perceived trustworthiness, and purchase intention are measured for each online platform separately. Two groups of data set undergo further analysis.

### Data Collection

The study is based on quantitative data analysis. The questionnaire survey is used as the primary tool of data collection. It was developed based on the previous studies and consisted of four parts following the conceptual model of the research: the first part covers the demographic information of the respondents, the second is related to the cultural dimensions (Hofstede, 1980), the third deals with trustworthiness dimensions (McKnight et al., 2002), and the fourth one is assessing the purchase intention online. The measurement of cultural dimensions on the individual level was applied to the analysis of both online platforms, while disposition to trust trustworthiness and purchase intention were measured separately for each online platform. A 5-point Likert scale was used in the questionnaire, ranging from 1 (strongly disagree) to 5 (strongly agree). The questionnaire was created in English and later translated into Russian. The pilot version of the questionnaire was presented to a group of respondents to ensure all the questions were easy to comprehend. All their comments and remarks were taken into consideration. After this, the modified version of the questionnaire was presented to the respondents.

The data for the analysis were collected through both an online survey platform and an offline distributed questionnaire to ensure sample representativeness and to cover different population groups. To collect the survey online, first, the questionnaire was compiled on the online platform Yandex.forms, and then the link to the questionnaire was randomly distributed using various social media platforms. The respondents who filled out the offline questionnaire were presented with the paper version. The data were collected from August 2019 to January 2020 and were further summarized and analyzed using AMOS SPSS 24. In total, 510 questionnaires were collected, and later, 46 were deleted as incomplete.

## Results

### Descriptive Statistics

Participants of the survey are Russian citizens of different age groups, which ensures the sample's representativeness compared to the majority of the surveys using students as a sample. However, in the e-commerce environment, it arguably reflects the overall tendency as the younger population generally demonstrates a higher ability to adjust to changes and accept new technologies. In the current sample, 57.3% of the respondents are older than 40 years. The summary of the descriptive statistics is presented in Table 1.

**Table 1:** Descriptive statistics.

**Variable**	**Items**	**Frequency**	**Percent, %**
Gender	Male Female	234 210	52.7 47.3
Age	18–25 26–30 31–40 41–50 51+	135 69 54 70 116	30.4 15.5 12.1 15.7 26.1
Frequency of online purchase	Daily Several times/week Several times/month Several times/year Less than once a year	13 34 124 137 136	3 7.6 27.9 30.9 30.6
Educational background	High school College Specialist (5 years) Undergraduate Postgraduate	72 86 149 56 81	16.2 19.3 33.5 12.6 18.2

*Source: Calculated by the authors*.

### Data Validation

Further data underwent multistep analysis. The measurement model was created in AMOS 24.0. In total, five measurements with low factor loading were removed. The re-specified model showed an acceptable model fit for two data sets subjected to the analysis. In order to validate the model, several indexes should be taken into consideration. Jaccard and Wan (1996, p. 87) recommend using at least three different tests to reflect different criteria. The model fit is presented in Table 2.

**Table 2:** Model fit.

	**Aliexpress**	**Ebay**	**Acceptable model (Schumacker and Lomax, 2004: 82)**
CMIN/DF	1.560	1.960	≤2
GFI	0.942	0.0.927	≥0.90
RMR	0.033	0.034	Closer to 0
CFI	0.978	0.966	≥0.90
RMSEA	0.036	0.047	≤0.05

*CMIN, Contrast Media-Induced Nephropathy; DF, Degree of Freedom; GFI, goodness of fit index; CFI, Comparative fit index; RMR, Root Mean Square Residual; RMSEA, Root Mean Squared Error of Approximation. Source: Calculated by the authors using AMOS 24.0*.

After the assessment of model fit, another important indicator, statistical power, was measured. Measurement of the statistical power is necessary to estimate the possibility of employing good and bad theory-implied constraints and specifications (Cohen, 1988, 1992). The study employs Structural equation modeling (SEM) for the analysis; therefore, to calculate statistical power, root mean square error approximation (RMSEA) index of the overall model fit is used (MacCallum et al., 1996). An online code generator was used to compute statistical power. Both models show relatively high indicators: 0.999 for the Aliexpress dataset and 0.996 for the eBay dataset (Preacher and Coffman, 2006).

At the preliminary stage of the analysis, constructs validity, and reliability were assessed (Table 3). Composite reliability (CR) was used to estimate the construct reliability (Brunner and Suss, 2005). The CR indicators of the constructs fall within the acceptable threshold of >0.60. Factor loadings of the constructs were assessed, demonstrating numbers of >0.50. Following Anderson and Gerbing and Anderson (1988), this demonstrates convergent validity. To further ensure validity, average variance extracted (AVE) was computed as well. Obtained values were higher than 0.50, which, according to Hair et al., 2010, indicates good construct validity. Discriminant validity is presented in Table 4 with squared AVE higher than the squared correlation of the variables.

**Table 3:** Construct validity and reliability.

	**Aliexpress**		**eBAy**	
	**CR**	**AVE**	**CR**	**AVE**
UAI	0.8598	0.6205	0.8596	0.6205
LO	0.9318	0.5287	0.9322	0.5286
DT	0.8377	0.5363	0.8927	0.6164
AB	0.9341	0.7002	0.9444	0.7058
INT	0.9266	0.7295	0.8882	0.5903
BEN	0.9017	0.6595	0.8891	0.7396
PI	0.8667	0.6471	0.9307	0.6443

*UAI, uncertainty avoidance; LO, long-term orientation; DT, trust disposition; AB, ability; INT, integrity; BEN, benevolence; PI, purchase intention; CR, composite reliability; AVE, average variance extracted*.

*Source: calculated by the authors*.

**Table 4:** Discriminant validity.

	**UAI**	**LO**	**DISP**	**AB**	**INT**	**BEN**	**PI**
**Aliexpress**							
UAI	**0.7877**						
LO	0.0102	**0.7271**					
DISP	0.0279	0.0193	**0.7322**				
AB	0.0190	0.0018	0.0713	**0.8367**			
INT	0.0077	0.0021	0.0702	0.1624	**0.8541**		
BEN	0.0001	0.0094	0.0202	0.1176	0.4356	**0.843**	
PI	0.0028	0.0002	0.0202	0.0007	0.0276	0.0369	**0.8120**
**eBay**							
UAI	**0.7877**						
LO	0.0102	**0.78**					
DISP	0.0246	0.0292	**0.7851**				
AB	0.0077	0.0044	0.0086	**0.8401**			
INT	0.0008	0.0010	0.0576	0.1832	**0.7683**		
BEN	0.0036	0.0022	0.0038	0.2088	0.7225	**0.8026**	
PI	0.0014	0.0066	0.0038	0.0059	0.1018	0.0835	**0.8599**

*UAI, uncertainty avoidance; LO, long-term orientation; DT, trust disposition; AB, ability; INT, integrity; BEN, benevolence; PI, purchase intention; CR, composite reliability; AVE, average variance extracted*.

*Square root of AVE on diagonal, correlation of constructs are in columns under AVE*.

*Source: calculated by the authors*.

*Bold values are statistically significant indicators*.

In order to assess the likelihood of common method bias, a common latent factor method was used (Serrano et al., 2018). There were no significant variances observed during the comparison of the standardized regression weight with and without common latent factor.

### Hypotheses Testing

Structural equation modeling is used to assess the hypotheses. The study uses path analysis to evaluate the casual paths as well as to identify the mediation effect in the model. The model analyzes two data sets to test the hypotheses. Table 5 demonstrates the results of the hypothesized effect for the Aliexpress and eBay datasets. The results show a significant effect of UAI (*p* < 0.01) on trust disposition, confirming H1 for both data sets. A statistically significant effect of long-term orientation on trust disposition is observed in the Aliexpress (Std. β = 0.128, *p* < 0.05) and eBay data sets (Std. β = 0.158, *p* < 0.01). Therefore, H2 is confirmed for both sets of data. H3 stating the relationship between trust disposition and ability is confirmed for Aliexpress (Std. β = 0.301, *p* < 0.001), while for eBay, this relationship does not show statistical significance, rejecting the hypothesis. The effect of trust disposition on benevolence was found to be statistically significant for both data sets: Aliexpress (Std. β = 0.294, *p* < 0.001) and eBay (Std. β = 0.148, *p* < 0.01). H4 is supported. H5 assessing the relationship between trust disposition and integrity is supported for both Aliexpress (Std. β = 0.297, *p* < 0.001) and eBay (Std. β = 0.148, *p* < 0.001). The effect of uncertainty avoidance on ability, assessed in H6, shows statistical significance for both Aliexpress (Std. β = 0.193, *p* < 0.001) and eBay (Std. β = 0.117, *p* < 0.05). H7 is supported only for Aliexpress (Std. β = 0.142, *p* < 0.01). The effect of uncertainty avoidance on benevolence does not show statistical significance in either data set. Therefore, H8 is rejected. The effect of long-term orientation on the dimensions of trustworthiness is not significant in any analyzed data sets. H9–H11 are rejected. Ability does not show a statistically significant effect on purchase intention for Aliexpress or eBay. Consequently, H12 is rejected. A statistically significant effect of integrity on purchase intention is observed for eBay (Std. β = 0.418, *p* < 0.01), supporting H13 for eBay. H14, assessing the effect of benevolence on purchase intention, is supported for Aliexpress (Std. β = 0.190, *p* < 0.05). The effect of trust disposition on purchase intention is significant both for Aliexpress (Std. β = −0.233, *p* < 0.001) and for eBay (Std. β = −0.153, *p* < 0.05). H15 is supported for both sets of data.

**Table 5:** Results of hypothesized effect.

		**Aliexpress**			**eBay**		
**Hypothesis**	**Path**	**Std. β**	**C.R**.	***P***	**Std. β**	**C.R**.	***P***
H1	DISP <–UAI	−0.166	−3.080	0.002^**^	−0.150	−2.899	0.004^**^
H2	DISP <–LO	0.128	2.184	0.029^*^	0.158	2.781	0.005^**^
H3	AB <–DIS	0.301	5.375	^***^	0.088	1.623	0.105
H4	BEN <–DIS	0.294	5.155	^***^	0.148	2.741	0.006^**^
H5	INT <–DIS	0.297	5.194	^***^	0.270	4.604	^***^
H6	AB <–UAI	0.193	3.758	^***^	0.117	2.239	0.025^*^
H7	INT <–UAI	0.142	2.734	0.006^**^	0.055	0.987	0.324
H8	BEN <–UAI	0.047	0.905	0.365	0.085	1.629	0.103
H9	AB <–LO	−0.071	−1.288	0.198	0.060	1.053	0.292
H10	INT <–LO	−0.074	−1.333	0.183	−0.007	−0.121	0.904
H11	BEN <–LO	0.058	1.027	0.305	0.029	0.518	0.605
H12	PI <–AB	−0.023	−0.379	0.704	−0.096	−1.574	0.115
H13	PI <–INT	0.110	1.358	0.174	0.418	2.654	0.008^**^
H14	PI <–BEN	0.190	2.345	0.019^*^	−0.003	−0.022	0.983
H15	PI <–DISP	−0.233	−3.702	^***^	−0.153	−2.512	0.012^*^

Significant at *p < 0.05,

** *p < 0.01*,

*** *p < 0.001*.

*UAI, uncertainty avoidance; LO, long-term orientation; DT, trust disposition; AB, ability; INT, integrity; BEN, benevolence; PI, purchase intention*.

*Source: Calculated by authors by using AMOS 24*.

*Bold values are statistically significant indicators*.

### Mediating Effect of Disposition to Trust and Trustworthiness

As the primary goal of the current study is to analyze the interaction between the personal traits and their effect on purchase intention, for a deeper assessment, the datasets are tested for the direct, indirect, and total effects for each context. Bootstrap analysis is performed with bootstrap samples set to 2,000 and a bias-corrected confidence interval set at 95 to assess the direct, indirect, and total effects of the constructs on each other within the context. The bias-correlated percentile method is used to obtain bias-correlated confidence interval and significance test. Estimands presented in Table 6 were defined to assess specific mediation effects.

**Table 6:** Mediating effect.

	**Aliexpress**		**eBay**	
	**Estimate**	***P***	**Estimate**	***P***
UAI->BEN->PI	0.007	0.236	0.000	0.972
UAI->AB->PI	−0.003	0.683	−0.007	0.109
UAI->INT->PI	0.011	0.267	0.015	0.224
LO->BEN->PI	0.013	0.209	0.000	0.995
LO->AB->PI	0.002	0.461	−0.006	0.240
LO->INT->PI	−0.010	0.230	−0.003	0.819
UAI->DIS->BEN	**−0.039**	0.004^**^	**−0.016**	0.025^*^
UAI->DIS->AB	**−0.040**	0.005^**^	−0.010	0.151
UAI->DIS->INT	**−0.039**	0.005^**^	**−0.028**	0.005^**^
UAI->DIS->PI	**0.028**	0.006^**^	**0.015**	0.021^*^
LO->DIS->BEN	**0.048**	0.017^*^	**0.027**	0.022^*^
LO->DIS->AB	**0.050**	0.016^*^	0.016	0.136
LO->DIS->INT	**0.048**	0.018^*^	**0.047**	0.004^**^
LO->DIS->PI	**−0.035**	0.011^*^	**−0.025**	0.018^*^

Significant at *p < 0.05,

** *p < 0.01*.

*UAI, uncertainty avoidance; LO, long-term orientation; DT, trust disposition; AB, ability; INT, integrity; BEN, benevolence; PI, purchase intention*.

*Source: Calculated by authors by using AMOS 24*.

*Bold values are statistically significant indicators*.

No statistically significant effect of the dimensions of the national culture on purchase intention mediated by the dimensions of trustworthiness is observed in either of the two data sets. The effect of uncertainty avoidance on ability mediated by the disposition to trust (UAI->DIS->AB) shows statistical significance for Aliexpress (−0.040; *p* < 0.01). Following Zhao et al. (2010) mediator effect classification, the mediator effect of uncertainty avoidance on ability can be classified as competitive mediation. The effect of the uncertainty avoidance on integrity mediated by the disposition to trust (UAI->DIS->INT) is found to be significant for both data sets, with a stronger negative effect for Aliexpress (−0.039, *p* < 0.01) when compared to eBay (−0.028, *p* < 0.01). For Aliexpress, it can be classified as competitive mediation; both direct and indirect effects exist, although pointing at different directions (Zhao et al., 2010). However, for eBay, indirect-only mediation is observed. The effect of the uncertainty avoidance on benevolence mediated by the disposition to trust (UAI->DIS->BEN) showed significance for both data sets, with a stronger negative effect for Aliexpress (−0.039, *p* < 0.01) when compared to eBay (−0.016, *p* < 0.05). Indirect-only mediation is observed for both sets of data. The effect of uncertainty avoidance on purchase intention mediated by the disposition to trust (UAI->DIS->PI) shows a positive statistically significant effect in both data sets, where the effect for Aliexpress (0.028, *p* < 0.01) is stronger when compared to eBay (0.015, *p* < 0.05). The effect of uncertainty avoidance on purchase intention is indirect-only.

The long-term orientation shows a positive significant effect on ability (LO->DIS->AB) for Aliexpress (0.05, *p* < 0.05). Indirect-only mediation is observed in the data set. The effect of long-term orientation on integrity (LO->DIS->INT) shows statistical significance for both Aliexpress data set (0.048, *p* < 0.05) and eBay (0.047, *p* < 0.01) with indirect-only mediation. The positive effect of long-term orientation on benevolence mediated by the disposition to trust (LO->DIS->BEN) is observed in both data sets, with a stronger effect for Aliexpress (0.048, *p* < 0.05) when compared to eBay (0.027, *p* < 0.05). Indirect-only mediation is observed for both Aliexpress and eBay. Negative effect of long-term orientation on purchase intention (LO->DIS->PI) is observed in both data sets, with stronger negative effect in Aliexpress (-0.035, *p* < 0.05) when compared to eBay (−0.025, *p* < 0.01). An indirect-only type of mediation is observed for both sets of data.

## Discussion and Conclusion

The study attempts to assess the interaction of the customer's personal traits and their effect on purchase intention in different contexts. Two dimensions of the national culture measured at the individual level, disposition to trust e-commerce platforms, perceived trustworthiness related to particular platforms, and purchase intention are assessed to answer the research question. Based on the geography of the consumption and the country-of-origin factor, disposition to trust, trustworthiness dimensions, and purchase intention are measured regarding Aliexpress (Chinese) and eBay (the US) data sets.

A comparison of the assessed hypotheses gives a clear understanding of the fact that the personal traits of the customers act differently within the context of the platforms. Their influence on purchase intention across platforms varies. The assessed effect of the dimensions of national culture toward the trust disposition was found to be different for the two marketplaces. So, the direct effect of uncertainty avoidance has a stronger negative effect on the trust disposition for the Aliexpress sample. On the contrary, the positive direct effect of long-term orientation on trust disposition is stronger for eBay. Both findings can be explained by a stronger country-of-origin image of eBay and confirmed by the existing analytical data signifying that customers tend to purchase more expensive and durable goods from the western platforms. Confirming previous findings (Gefen and Straub, 2004; Teo and Liu, 2007; Kim et al., 2008; Hallikainen and Laukkanen, 2018), trust disposition showed a positive effect on trustworthiness dimensions, except the effect of trust disposition on ability for eBay, which did not show statistical significance. Meanwhile, the effect of trust disposition on trustworthiness dimensions was stronger for Aliexpress. The effect of uncertainty avoidance on ability showed significance, confirming previous findings (Hwang and Lee, 2012; Hallikainen and Laukkanen, 2018). Proving the research supposition that personal traits interact differently depending on the context, the Aliexpress dataset shows direct and mediated effects, while only direct effect is observed for eBay. Both direct and mediated effects of uncertainty avoidance on integrity are observed for Aliexpress, while only indirect effect is observed for eBay, confirming previous findings (Hwang and Lee, 2012). The mediated effect is foun to be negative for both sets of data, with a stronger indicator for Aliexpress. This finding may signify that customers are less assured that Aliexpress would act according to the agreement than eBay. The hypothesis related to the effect of uncertainty avoidance on benevolence is not confirmed, proving previous findings. However, a mediated effect between uncertainty avoidance and benevolence is found for both marketplaces. The relationship has a negative nature with a stronger negative effect for Aliexpress. This finding may reflect that consumers' doubts about Aliexpress acting in their interests are higher than for eBay. The effect of long-term orientation on trustworthiness dimensions showed indirect-only mediation. Confirming previous findings (Hallikainen and Laukkanen, 2018), it is found that long-term orientation has the strongest effect on ability (confirmed in Aliexpress data set), followed by integrity and benevolence. The mediated effect of long-term orientation on benevolence is much higher for Aliexpress than for eBay, while the difference in the effect of long-term orientation on integrity is small.

Although some studies show that ability significantly affects purchase intention (Gefen, 2000; Colquitt et al., 2007; Lu et al., 2010; Hwang and Lee, 2012), current research did not find a statistically significant relationship between ability and purchase intention. The effect of benevolence on purchase intention is observed only for Aliexpress, proving some previous findings (Colquitt et al., 2007). The effect of integrity on purchase intention is observed only for the eBay data set, supporting previous findings (Gefen, 2000; Colquitt et al., 2007; Hwang and Lee, 2012). The direct effect of disposition to trust is observed for both data sets confirming previous findings (Gefen and Heart, 2006). The findings show that this relationship has negative nature with stronger indicators for Aliexpress.

Based on the analysis conducted, it can be concluded that purchase intention is affected by various factors. It is proven that in a different context, the effect of these factors varies. After assessing direct and mediated effects of the constructs, it can be concluded that context moderates the interaction of variables and affects the customers' behavioral outcome. The difference in the analyzed cultures can explain the mismatch with the previous findings. The research findings point out the significance of the customers' personal traits, which cannot be generalized in application to a specific context.

## Contribution and Implication

The current study contributes to the existing literature on consumers' online behavior in several ways. First, it adds to the cultural research in the e-commerce environment, which is limited to a specific group of countries, the majority of which do not obtain a high level of UAI, which diminishes the significance of trust in the acceptance and usage of e-commerce. Second, it addresses a country with a developing e-commerce infrastructure where the acceptance of e-commerce practices is at a critical stage. Meanwhile, the analysis of e-commerce practices in such countries is limited.

The study advocates that the behavioral outcome differs in various contexts. Consequently, in e-commerce environments, purchasing intention varies depending on the platform. It also contributes to the theoretical knowledge about the Russian e-commerce environment and can be used for a similar analysis in other countries and expanded for a more comprehensive analysis.

The research benefits the practitioners operating in the international e-commerce environment, facilitating cross-border e-commerce by providing a more extensive image of customers' personal traits and their effect on purchase behavior. To date, the significance of trust in the development of e-commerce is crucial. A detailed understanding of which dimension of trust can affect the potential purchase intention ensures possible success or failure. Thus, e-commerce is closely related to a high level of uncertainty, particularly for the countries where e-commerce is a comparatively new practice In order to minimize its level, managers and marketers should understand their customers' fears and preferences. The effect of perceived integrity on purchase intention, in the case of the current study relevant for eBay, may trigger such issues as doubts about the quality of the product. The effect of perceived benevolence is related to such issues as the disbelief that the platform operates in their interest, consequently, affecting payment intention. Perceived ability defines customers' skill to believe that the shop or platform can fulfill their duty and, consequently, can reduce the level of uncertainty, which is crucial for trust building (Gefen, 2002). Cross-border e-commerce practitioners should be able to incorporate the understanding of their customers in their praxis. Skillful operation with the customers' culture and trustworthiness dimensions may facilitate long-term trust, reducing the level of uncertainty and transforming potential buyers into loyal customers.

## Data Availability Statement

The raw data supporting the conclusions of this article will be made available by the authors, without undue reservation.

## Ethics Statement

The studies involving human participants were reviewed and approved by the study involving human participants was reviewed by the Research Ethics Committee of China University of Geosciences(Wuhan). The patients/participants provided their written informed consent to participate in this study.

## Author Contributions

AT and SC: research design. AT, DK, MK, and HA: data collection. AT theoretical framework development. JH and AT: data analysis. SC and DK: manuscript revision. All authors discussed the results and contributed to the final manuscript.

## Conflict of Interest

The authors declare that the research was conducted in the absence of any commercial or financial relationships that could be construed as a potential conflict of interest.

## Publisher's Note

All claims expressed in this article are solely those of the authors and do not necessarily represent those of their affiliated organizations, or those of the publisher, the editors and the reviewers. Any product that may be evaluated in this article, or claim that may be made by its manufacturer, is not guaranteed or endorsed by the publisher.

## Footnotes

^1^https://yandex.ru/company/researches/?type=researches&tag=ecommerce [accessed June 5, 2020].

^2^https://press.aliexpress.ru/page18635284.html [accessed May 25, 2021].

^3^https://www.ebayinc.com/stories/news/ebay-expands-global-presence-new-russian-language-site-and-first-ever-ad-campaig-1/ [accessed May 25, 2021].
