# Supplementary material for: How Culture and Trustworthiness Interact in Different E-Commerce Contexts: A Comparative Analysis of Consumers' Intention to Purchase on Platforms of Different Origins
Source: Front Psychol. 2021 Oct 5;12:746467. doi: 10.3389/fpsyg.2021.746467 (PMC8523785; doi:10.3389/fpsyg.2021.746467)
Supplement: Supplementary file 1 [file Data_Sheet_1.PDF]

Supplementary Material

1. Questionnaire sample

Part 1: General Information

Please tick in the appropriate box

Gender Male Female

Age (In Years) 18- 30 31 - 40 41 - 50 50 above

Education high school college specialist undergraduate postgraduate

How often do you buy goods online?

- Daily
- Several times/week
- Several times/month
- Several times/year
- Less than once a year

Part 2: CVSCALE

Please select the most appropriate number for you (1 disagree - 5 strongly agree)

	**1 2 3 4 5**
**Uncertainty avoidance**	**1 2 3 4 5**
1. It is important to have instructions spelled out in detail so that I always know what I'm expected to do<br>2. It is important to closely follow instructions and procedures.<br>3. Rules and regulations are important because they inform me of what is expected of me.<br>4. Standardized work procedures are helpful.	
**Long-term orientation**	**1 2 3 4 5**
It is important for me:<br>1. Personal steadiness and stability.<br>2. Long-term planning.<br>3. Working hard for success in the future.	

Source: *adopted from Hofstede (1980)*

Part 3: Trust

Range each sentence from 1 to 5 (1-strongly disagree / 5 - strongly agree) for each e-commerce platform in the table.

**Trust dimension**	**Aliexpress**	**eBay**
**Disposition of trust**	1 2 3 4 5	1 2 3 4 5

Trust dimension	Aliexpress	eBay
1. I generally trust online platform<br>2. I tend to count on online platform.<br>3. I generally have faith in humanity.<br>4. I feel that the platform is generally reliable.		
**Ability**	1 2 3 4 5	1 2 3 4 5
1. They are competent and effective.<br>2. They perform their role of giving advice very well.<br>3. Overall, they are capable and proficient.<br>4. In general, they are very knowledgeable about their own merchandise.		
**Integrity**	1 2 3 4 5	1 2 3 4 5
1. They are truthful in their dealings with me.<br>2. I would characterize them as honest.<br>3. They would keep their commitments.		
**Benevolence**	1 2 3 4 5	1 2 3 4 5
1. I believe that they would act in my best interest.<br>2. If I required help, they would do their best to help me.<br>3. They are interested in my well-being, not just their own.		

Source: *adopted from McKnight et al. (2002)*

Part 4: Purchase intention to buy
please rate from 1 to 5 (1 strongly disagree - 5 strongly agree)

	Aliexpress (1 2 3 4 5)	eBay(1 2 3 4 5)
1. While browsing the platform I'm willing to make a purchase		
2. I prefer to buy goods online		
3. I consider buying online is simple and convenient		
